# Disease-directed engineering for physiology-driven treatment interventions in neurological disorders

**DOI:** 10.1063/1.5117299

**Published:** 2019-10-23

**Authors:** Thomas Wood, Elizabeth Nance

**Affiliations:** 1 Department of Pediatrics, University of Washington, Seattle, 98195 Washington, USA; 2 Institute for Human and Machine Cognition, Pensacola, 32502 Florida, USA; 3 Department of Chemical Engineering, University of Washington, Seattle, 98195 Washington, USA; 4 Department of Radiology, University of Washington, Seattle, 98195 Washington, USA; 5 Center on Human Development and Disability, University of Washington, Seattle, 98195 Washington, USA

## Abstract

Neurological disease is killing us. While there have long been attempts to develop therapies for both acute and chronic neurological diseases, no current treatments are curative. Additionally, therapeutic development for neurological disease takes 15 years and often costs several billion dollars. More than 96% of these therapies will fail in late stage clinical trials. Engineering novel treatment interventions for neurological disease can improve outcomes and quality of life for millions; however, therapeutics should be designed with the underlying physiology and pathology in mind. In this perspective, we aim to unpack the importance of, and need to understand, the physiology of neurological disease. We first dive into the normal physiological considerations that should guide experimental design, and then assess the pathophysiological factors of acute and chronic neurological disease that should direct treatment design. We provide an analysis of a nanobased therapeutic intervention that proved successful in translation due to incorporation of physiology at all stages of the research process. We also provide an opinion on the importance of keeping a high-level view to designing and administering treatment interventions. Finally, we close with an implementation strategy for applying a disease-directed engineering approach. Our assessment encourages embracing the complexity of neurological disease, as well as increasing efforts to provide system-level thinking in our development of therapeutics for neurological disease.

## I. INTRODUCTION

There are more than 600 diseases of the nervous system that impact normal function of the brain, spine, or the nerves that connect them.^1^ Acute neurological injury includes strokes and other conditions that result in cerebral hypoxia-ischemia (HI) such as cardiac arrest, as well as traumatic brain injury (TBI). In the U.S., TBI accounts for 2.5 million emergency room visits every year, and up to 5.3 million people are thought to be living with TBI-related disability.^2^ Worldwide, more than 6 million people die from a stroke each year.^3^ Importantly, both TBI and cerebral HI have long-term ramifications even though the neurological event is acute. Chronic neurological diseases include Alzheimer's disease (AD), multiple sclerosis (MS), Parkinson's disease (PD), amyotrophic lateral sclerosis (ALS), Huntington's disease, a number of cancers, neuromuscular disease, epilepsy, autism, depression, bipolar disorder, and schizophrenia. Chronic neurological disease afflicts more than 50 million Americans each year and makes up 8% of the global health burden.^4^ With 10 000 Americans turning 65 each day,^5^ the burden of neurological morbidity will only continue to increase as the population ages.

While there have long been attempts to develop therapies for both acute and chronic neurological diseases, no current treatments are curative. Current market reports show almost 600 medicines in development to prevent or treat a variety of neurological disorders,^6^ but many pharmaceutical companies have become increasingly divested from neuroscience research efforts.^7^ This is often because the cost and time scale for new therapeutics to reach target patient populations are high. Using AD as an example, the total cost of AD drug development is estimated at $5.6 billion, spanning an average 13-year process from preclinical studies to approval by the Food and Drug Administration (FDA).^8^ However, the cost and time scale are not the only, or even most significant, challenge. The failure rate of AD drug development for disease-modifying therapies is 99%.^8^ Interestingly, while any therapeutic entering clinical development will have demonstrated evidence of efficacy and safety in preclinical models, therapeutics still face a greater than 90% chance of failing due to the lack of clinical efficacy or the presence of side effects that are intolerable to the patient.^9^ Failure rates for neurological disease therapeutics remain disproportionately high compared with other disease areas,^10^ with most failures coming in late stage clinical trials.^11^ Indeed, the most recent significant pharmaceutical step-change in neurology was almost three decades ago in 1991, when Sumatriptan was approved for the treatment of migraines. The field therefore critically needs a better understanding of brain disease and therapeutic processes, and an improved ability to translate these findings into effective biomarkers and therapeutics.

**FIG. 1.**
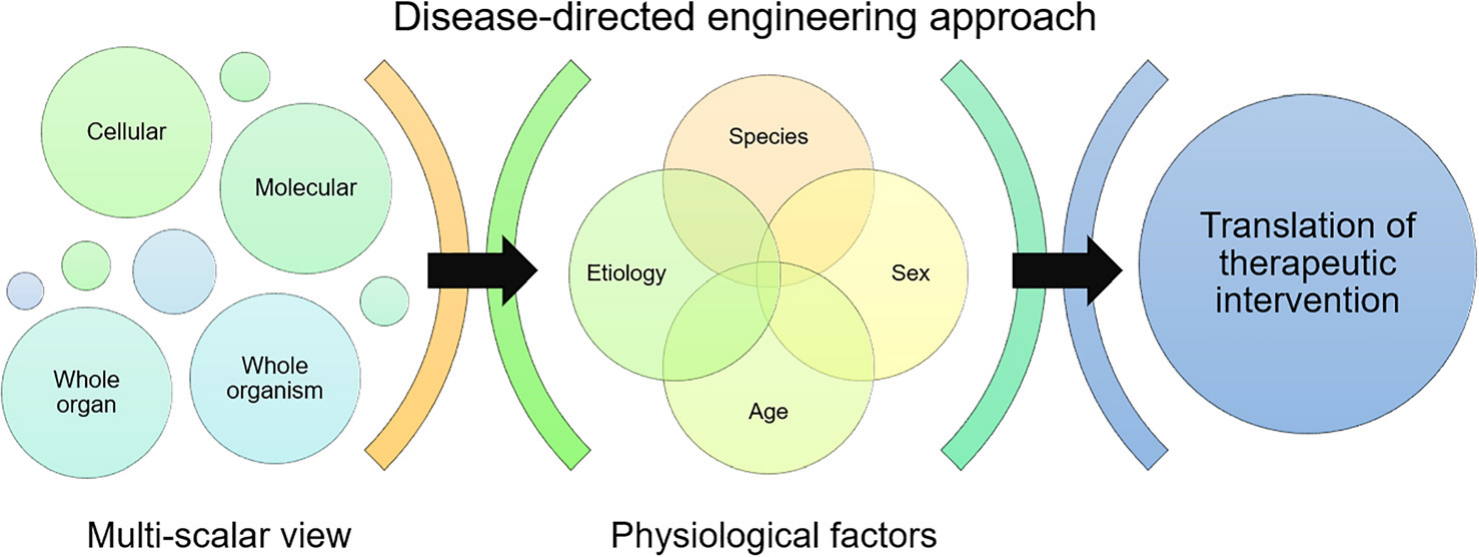
A disease-directed engineering approach takes a multiscalar view of assessing treatment outcomes, and accounts for key physiological factors that could affect translation of therapeutic interventions into clinical application.

The standard approach to studying disease is often reductionist, and our focus on individual molecules or pathways in a disease system has more often than not failed to produce therapies for that disease.^12^ It is important to remember that diseases are caused by a combination of perturbations to a complex system, and similar disease phenotypes might be caused through different pathways in different patients. To successfully close the gap in need for effective therapeutics for neurological disease, an engineering approach can, and should, play a critical role. Hence, our perspective is that physiology-centered treatment strategies studied in a multiscalar way should drive the engineering of therapeutic interventions (Fig. 1). However, engineering therapeutics for complex disease requires incorporation of critical aspects of the underlying physiology and pathology. We highlight open challenges where an understanding of basic physiology can direct how, when, and with what we should intervene in the treatment of neurological disease. We first address the need to account for the effect of age and sex, species differences, and systemic vs local physiological differences in the preclinical-to-clinical translation process. We dig into why the timing of physiological changes should matter in guiding our therapeutic design and intervention. We then highlight a nanotherapeutic success in physiological-driven treatment intervention for developmental brain injury, and close with a perspective on moving forward using the concept of engineering high-level vs mechanistic intervention approaches for treating neurological disease.

## II. NORMAL PHYSIOLOGICAL VARIATIONS SHOULD GUIDE EXPERIMENTAL DESIGN

In 2014, the National Institutes of Health (NIH) implemented guidelines for researchers to address biological variables in their preclinical study plans and outcome assessments.^13^ The goal of this guideline change was to enable researchers to account for confounding and selection biases, as well as to better capture the “real world” in order to enhance the reproducibility and translatability of biomedical research. We emphasize the importance of species-based differences in physiology that will influence the translation process, especially in our evaluation of the therapeutic effect. We also discuss the need to think beyond the local physiology of the target site and consider the systemic physiology that will influence a therapeutics' success.

### A. Age-based physiological differences

Brain development is modular in both structure and function, which introduces opportunities as well as limitations in age-matching preclinical animal studies to humans.^14^ At the macroscopic level, aging of the brain results in volume and brain weight loss with each decade after age 40,^15^ a decrease in cerebral blood flow (CBF),^16^ and shrinking of the gray matter.^17^ Childhood brain development represents the other end of the spectrum. Most current therapeutics for children are often extrapolated from studies in adult animals or humans.^18^ However, children present a range of growth and developmental phases, with physiologic and cognitive changes that are distinct from adults. These changes can alter pharmacokinetics (PK) and pharmacodynamics of therapeutics, and result in a different response to both active drug and formulation excipients.^19^

We have several existing examples of where therapeutic development does not translate well from children to adults or adults to children, based on the differences in physiology. In response to HI, therapeutic hypothermia (TH), or selective cooling of the head or body, has long been a treatment approach to reducing mortality and morbidity. TH is currently the standard of care for term newborns with hypoxic-ischemic encephalopathy (HIE), and has been demonstrated to significantly reduce the risk of death and neurodevelopmental disability,^20^ although approximately 50% still have poor outcomes.^21–24^ However, the evidence for TH in adult acute ischemic stroke not caused by cardiac arrest is less convincing.^25^ After adult out-of-hospital cardiac arrest, ensuring normothermia and preventing pyrexia may in fact be as beneficial as TH.^26^ The TBI field has seen similar lack of translation across all ages for TH treatments. Though a recent meta-analysis performed in 2017 suggested that TH provides an 18% reduction in mortality and 35% improvement in neurologic outcome for adults,^27^ this was highly contested by those involved in the field, as all the high-quality trials in both adult and pediatric TBI have failed to show benefit.^28^

Another example of age-dependent benefits is superoxide dismutase (SOD), an endogenous enzyme that converts superoxide into hydrogen peroxide, and is reduced after injury, resulting in excess superoxide production. Treatment with SOD in preclinical models of TBI and ischemic stroke has resulted in decreased brain edema and improved motor function.^29,30^ However, beneficial results have been less clear in neonatal models of acute brain injury. For instance, while SOD overexpression is neuroprotective in a rodent model of adult stroke,^31^ it may exacerbate injury in the neonatal brain.^32^ Similarly, exogenous poly(ethylene glycol) (PEG)-conjugated SOD in neonatal HIE displays a U-shaped curve of neuroprotection, with benefits loss at higher doses.^33^ This is likely to be due to a relative underexpression of catalase in the developing brain, resulting in accumulation of hydrogen peroxide.^32,34^ Therefore, if one were to engineer targeted antioxidant therapies, the level of development and responsiveness of the antioxidant system at the target age of intervention would need to be accounted for in therapeutic studies.

### B. Sex as a biological variable

In a 2011 analysis of peer-reviewed literature in neuroscience research, the authors found that sex was not reported in 80% of articles,^35^ which is an alarming statistic considering the abundance of clinical data showing sex-based differences in prevalence and outcomes of most neurological diseases. The NIH response in 2014 was to mandate that investigators studying neurological disease must design studies that allow for examination of differences across sex, or provide justification for not doing so. There are opposing thoughts on the validity of this approach in improving translation,^36,37^ but it is worth highlighting the need of accounting for sex in preclinical research for the design and evaluation of therapeutic interventions.

There are well-established differences in the clinical literature for how diseases manifest and progress in males and females.^38^ Compared to females, male infants are more vulnerable to a perinatal insult and suffer more long-term cognitive deficits compared to females with comparable injury.^39,40^ Males are two times more likely to experience prenatal anoxia, hemorrhage, and infection, and almost two times more likely to suffer cerebral birth trauma.^41^ Males are also more susceptible to neurodevelopmental disorders including intellectual disability, autism spectrum disorder (ASD),^42^ and attention-deficit activity disorder.^43^ In the case of pediatric TBI, males are two times more likely to sustain a TBI than females, yet mortality rates are the same compared to females.^44^ Interestingly, females have a longer stay in the hospital and a trend toward worse outcomes, even after controlling for injury severity.^45^ Females also experience higher rates of concussion,^46,47^ after controlling for higher rates of reporting of concussive symptoms in females compared to males.^48^ In adolescence and adulthood, major depressive disorder and anxiety disorders are almost twice as common in females as in males.^49,50^ Schizophrenia has a high incidence and earlier onset in males, and males also have a worse prognosis, largely thought to be due to more severe symptoms and a poorer response to antipsychotic medicines compared to females.^51,52^ If we look at neurodegenerative disease, there is a higher prevalence of AD in females above 65 years of age and greater cognitive deterioration.^53^ The prevalence of ALS is higher in males, and the onset is earlier in males, but there is a higher risk in postmenopausal women as well as worse survival for females with ALS.^54^ More females have MS compared to males, but MS has a faster progression in males.^55^ For PD, there is a higher incidence rate in males and a slower rate of decline in females.^56,57^ Acute brain trauma also shows sex-based differences. Stroke prevalence is higher in males compared to females.^58^ At 85 years of age and older, stroke is more common in females, although this is often attributed to the longer lifespan of females on average compared to males. Females under 75 have lower stroke mortality compared to males, an advantage that declines with age, but females also have less favorable outcomes and more severe physical disabilities.^59^

Differences in the outcomes of human neurological diseases are reflected mechanistically in animal models. In both adult and neonatal stroke models, females rely display more caspase-dependent cell death pathways, with males largely displaying caspase-independent pathways.^60–62^ These sex-based differences have been one factor thought to contribute to better outcomes in females after experimental therapies for neonatal HI brain injury.^39^ Studies *in vitro* and *in vivo* show that programmed cell death and autophagy of neurons follow different pathways in males vs females.^63–65^ Broad-acting disease pathways such as excitotoxicity, inflammation, and oxidative stress, all pathological hallmarks of most neurological disease, also show sex dependence. Microglia, cells that mediate inflammatory responses to disease or injury, show a region-specific increase in number in the neonatal period in males, but are greater in number in females in adulthood.^66^ There is some thinking that this switch is correlated with, or a potential reason for why human males have higher incidence of neurological disorders earlier in life and females suffer from neurological disease later in life.^67^ The ability to respond to or mitigate oxidative stress influences mitochondrial function, where male mitochondria have enhanced uptake capacity of calcium.^68^ Oxidative stress pathways follow a similar sex-dependent regulation. Neuronal nitrogen oxide species (nNOS) are greater in males than in females following cerebral ischemic injury,^69^ and so inhibition of nNOS is neuroprotective in males. However, in females, nNOS inhibition or knockout actually increases infarct volume, demonstrating that the therapeutic effect can be sex-dependent. Damage due to reactive oxygen species (ROS) damage is also higher in males in preterm infants^70^ and in TBI adults^71^ compared to females. Again, hormones are thought to play a key role here due to increases in antioxidant enzymes that can confer higher or more resilient defense systems. In the context of treating oxidative stress following brain injury, males may benefit more from antioxidant treatments, and therefore might necessitate further research to identify strong candidates for females suffering from injury.^72^

In light of these sex-based differences, it would be natural to think that the robustness of clinical data reflecting sex-based differences would be grounds to account for sex in preclinical research. Yet, the paucity of sex-balanced studies is almost unbelievable. In general preclinical research, male animals are used 3:1 or 4:1 compared to females, though this ratio is almost 6:1 in neuroscience, where the sex disparities are often huge.^35^ These disparities have remained significant up to 2016 when the most recent literature meta-analysis was performed.^37^ Ironically, one would think that accounting for basic variables such as expected lifespan of females vs males would be easy given that female mammals outlive their male counterparts in almost all species. However, the C57BL/6 mouse is one of the few mammals where the males outlive the females, thought to be due to genetic instability and altered antioxidant handling, and this strain makes up 80%–90% of rodents used in research.^73^

Capturing sex-based differences in animal models is challenging, but necessary. Yet concern around the subsequent increase in sample size to accommodate sex-based variability has certainly risen to the forefront of this conversation. This is particularly true in engineering therapeutics, where a multitude of control groups for treatment, delivery vehicle, and pathology are already incorporated in the study. In particular, this concern seemed to rise out of the anticipated variability introduced by hormonal fluctuations across the reproductive cycle in females, adding another layer to the already complex physiological equation. However, recent meta-analyses of over 10 000 trait measurements in mice,^74,75^ rats,^76^ and humans^75,77^ showed that variability was not greater in females compared to males, for any assessment. The use of females in research does not add variability any more so than the use of males,^74^ yet the absence of females in research studies can significantly reduce the capacity to translate preclinical therapeutic products to clinical implementation.

Sexually dimorphic disease pathology and outcome are likely to be even further exaggerated in humans compared to animals, with differences in size, body fat, activity (both type and amount), metabolism, energy demand,^78^ and responses to stress^79^ all influenced by the complex interplay between hormonal, genetic, immunological, and psychosocial factors.^80^ Even with the sex-based evidence in the clinical setting, and at the molecular and cellular level, it is still important to keep in mind the general inability of preclinical studies to capture the sex-gender association prevalent in humans. There are many sociocultural influences that are more strongly associated with the concept of gender rather than sex,^36^ which are not currently captured in animal models. Gender role expectations can influence reporting of symptoms, as well as subsequent data collected to diagnose a disease or track response to treatment.^81^ Implicit bias about gender has been demonstrated to influence diagnosis rates and treatment decisions.^82^ Each of these will confound the impact of an individual therapeutic being translated and tested in humans. Continued research on the fluidity between sex and gender and the impact on neurological function, pathology, and response to therapy will be necessary to further study in humans, and to the best of the fields' ability, in animal models with increasing rigor. Species variations extend beyond sex, and are discussed further in the Sec. II C, yet these challenges should not supersede the need to understand therapeutic implications in a sex-dependent manner. However, as with any other physiological factor, researchers should not treat sex in isolation or as more relevant than other sources of variance such as genetic, developmental, and environmental factors.

### C. Species variations in basic physiology

**FIG. 2.**
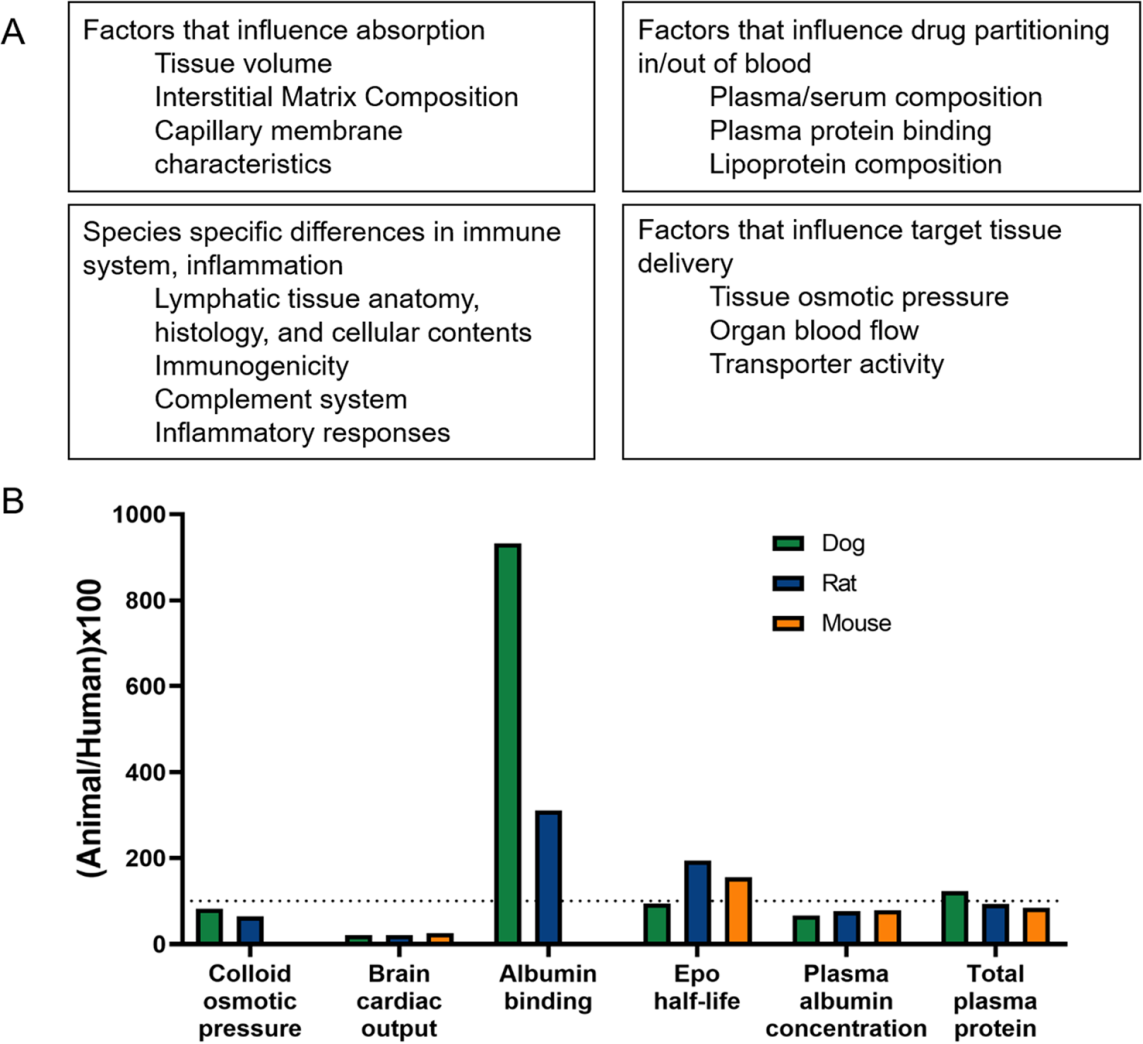
Summary of species differences that influence therapeutic outcome. (a) Factors that vary across species and influence drug absorption and partitioning, immune system and inflammatory responses, and delivery of a therapeutic to the target tissue. (b) Sample experimental values from various species, normalized to the human value and expressed as a percent, for a variety of physiological markers.^83^

Animal research has played a critical role in therapeutic development, yet animal models predict clinical efficacy with varying degrees of success. The human brain is complex, and there are evolutionary obstacles to developing animal models that adequately recapitulate human neurological disease and predict treatment efficacy. There is therefore a benefit to considering species differences as another of the underlying factors limiting translation of therapeutic interventions. Interspecies differences have been observed for many aspects that affect therapeutic efficacy, including drug absorption, distribution, and metabolism. Many of these species-based differences are summarized in Fig. 2, and reviewed extensively elsewhere.^83^

Studying multiple species can be beneficial, particularly to capture the heterogeneity of human disease through the variations in disease etiology and progression in different species. Importantly, insights gained from multiple species can help understand what can be generalized and what is species-specific. However, the choice of animal models is often constrained, whether that be due to traditions in the specific discipline or disease area, cost, expedience of the study, ethical considerations, political considerations, and institutional resources. In light of many of these reasons, rodents have become the most widely used preclinical model for neuroscience research. Mice represent an almost infinite space for genetic manipulation, which can allow for specific knockdown, knock-in, or knockout of key pathways to study disease and therapeutic effects. Additionally, mice are cost effective in many ways, for housing and maintenance, but also for drug development, where the smaller weight is advantageous when testing novel compounds, which are given on a dose-per-weight basis. Their small size is also advantageous for techniques like optogenetics.^84^ For understanding functional impact of disease and therapeutic interventions, rats are often cited as being a better model than mice due to a more complex behavioral repertoire.^85^ Rats possess a larger brain, which allows for easier surgical interventions and better spatial resolution in brain imaging, and are more resilient to stress caused by handling, which can confound results, particularly in the neuropsychiatric and neurodevelopmental fields. Rats have even been trained to sit still during imaging procedures without the need for anesthesia, which can interfere with brain activity.^86^ At the cellular and molecular levels, rats have similar levels and spatial distributions of many neurotransmitters and receptors as do humans.^84^ In addition to the aforementioned reasons, a recent improvement in the ability to perform genetic manipulation in rats is increasing the number of rats used in neuroscience animal research. However, a singular focus on rodent models could also be considered a contributor to the dramatic failure rate of therapeutics for neurological diseases.

Beyond rodents, nontraditional species offer additional advantages. Voles have demonstrated unique contributions to understanding of social interactions, including in an understanding of the role of oxytocin in pair-bond formation in mammals.^87^ Ferrets are an increasingly attractive species to model brain injury because of physical and developmental similarities to humans^88^ and complex social and cognitive behavior. The ferret has a ratio of white-to-gray matter that is similar to humans as well as a gyrencephalic cerebral cortex, unlike rodents and rabbits. Ferrets are born lissencephalic and develop gyrencephalic brains postnatally with white-matter maturation and cortical folding mirroring that of the third trimester of humans.^89^ Therefore, the utility of ferrets in modeling perinatal brain injury or neurodevelopmental disorders is high, especially with the increase in validation of assays and behavioral assessments to evaluate therapeutic effect.^88^ Ferrets have also been used extensively in immunology, especially influenza research, as their immunological responses more closely resemble those of humans than do rodents.^90^ Indeed, a seminal paper by Seok *et al.* in 2013 described how immunological responses to both trauma and endotoxemia in the mouse poorly mimic those of the human.^91^ While this assertion has been hotly contested, it highlights the dire need for a wider range of species to model inflammatory disease processes, which includes those present in the majority of neurological disorders. Larger animals such as sheep and pigs have also been proven as useful animal models for brain injuries and evaluation of therapeutic interventions, particularly in the treatment of neonatal HIE.^92^ Pigs and canines have routinely been used to model cardiac arrest-induced brain injury, given the anatomical similarities of the brain and cardiovascular system to humans.^93,94^ As our closest evolutionary ancestors, judicious use of nonhuman primates in research has also been highly beneficial, including in the investigation of neonatal HIE and disorders of aging.^95,96^

The number of animal models across species is growing, and it is necessary for those engineering or designing therapeutics to collaborate with a variety of researchers who provide a broader repertoire of animal models to continue to improve translation and species-dependent or independent aspects of those therapeutics. Regardless of the specific species used, a standard way of thinking is that meaningful comparisons can be achieved if data are based on functional outcomes. The Geroscience Network, a consortium of clinical and basic science aging researchers, has identified functional domains that are important for direct relevance of preclinical models to humans. These include cognitive, cardiovascular, inflammatory, metabolic functions, neuromuscular, and body composition and energetics.^97^ The neonatal HIE field has historically shown the most promise in terms of the breadth of animal models used to understand both the physiology of the disease and the response to therapies. The development of TH for term HIE encompassed case reports in asphyxiated human neonates several decades ago, mechanistic studies in both adult and newborn rats, detailed physiological studies in piglets, and the use of a fetal sheep model to develop the protocol now used in humans.^98^ Since the inclusion of TH in international resuscitation guidelines in 2010, mechanistic studies of TH in neonatal mice have been published, and nonhuman primates have been used to investigate TH in combination with the promising adjunctive therapy erythropoietin.^95,99^ We believe that this approach serves as a good model for developing therapeutics, particularly for acute neurological disorders, where studies in rodents are translated to rabbits, ferrets, or dogs followed by sheep and pigs, and finally nonhuman primates. This process could be considered a preclinical “funnel,” potentially including initial *in vitro* work with organotypic brain slice culture or brain organoids to screen therapeutics before applying them to rodents, and so on. Though a multispecies preclinical treatment funnel will increase the timeline associated with developing new therapies, success across multiple evolutionarily divergent species will dramatically increase the likelihood of translation to humans. Importantly, heterogeneity of metabolic and immune responses, as well as the necessary differences in how the disease is modeled in each species, will ensure that therapies emerging from the end of the funnel are sufficiently robust to have a high likelihood of success in the heterogeneous patient populations and clinical settings in which they will be applied. This approach will require close and careful collaboration between several laboratories who have expertise across the necessary model systems, which could be fostered by large collaborative grants from forward-thinking funding bodies.

## III. TIMING OF PATHOPHYSIOLOGICAL CHANGES MATTERS

It is one thing to know that underlying pathophysiology can drive treatment intervention and effect, but it is another to understand the timing in which those changes occur. Design of therapeutic interventions requires knowledge of the timing of when that therapeutic might be most useful, which requires understanding of the timing of underlying physiological changes that the therapeutic may target. In this section, we discuss acute physiological changes, changes that exist on the continuum from acute to chronic disease, and chronic physiological changes. Since most therapeutics are tested in the preclinical space, we close the section with a discussion on the challenges of modeling disease etiology and progression in the preclinical setting.

### A. Acute neurological disease

**FIG. 3.**
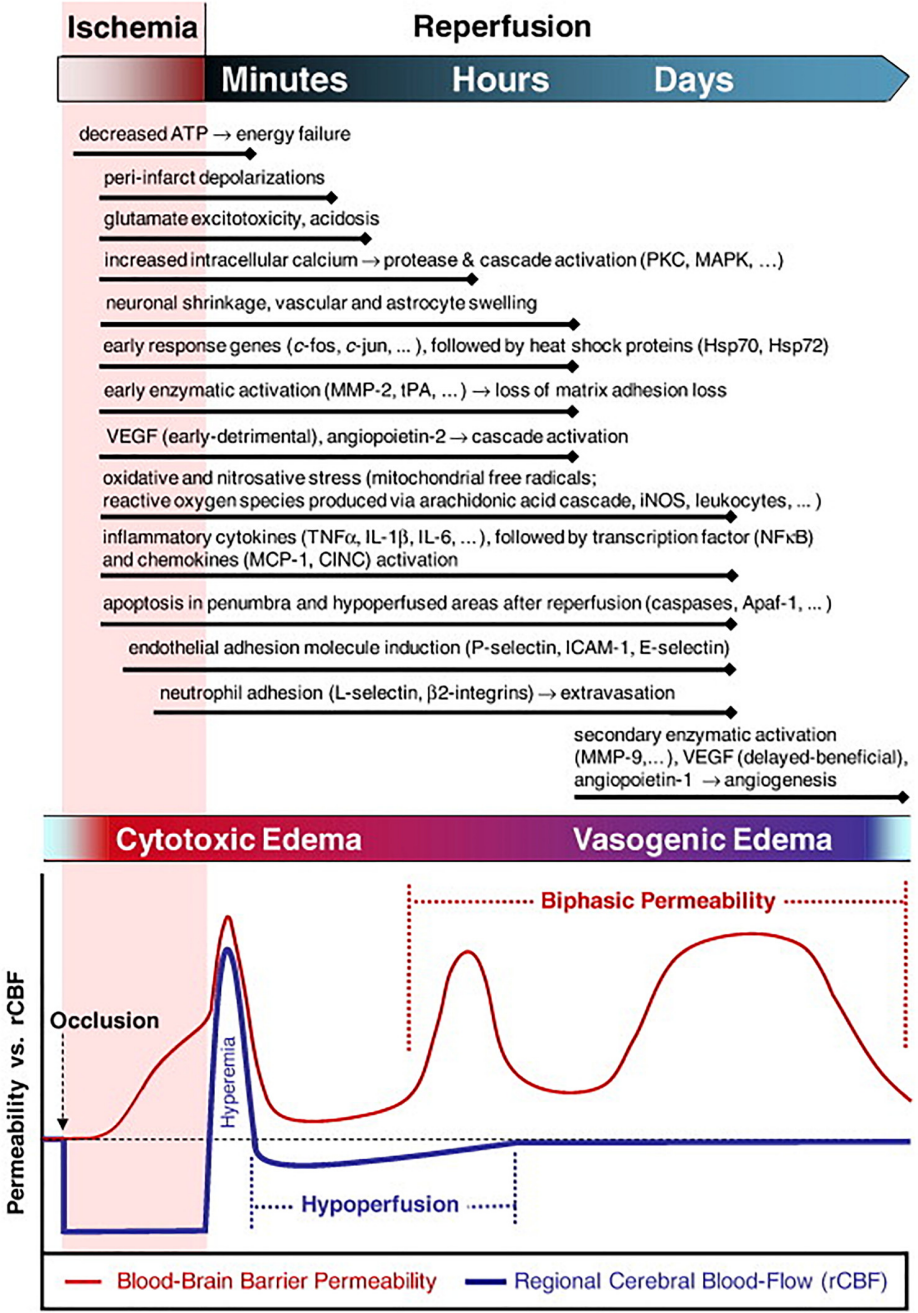
Phasic events of physiological aspects following ischemia and reperfusion in the brain. The current understanding of the timeline of edema, blood brain barrier permeability, cerebral blood flow, and inflammatory, cellular, and extracellular changes that occur following ischemia and during the reperfusion period. Reprinted with permission from K. E. Sandoval and K. A. Witt, “Blood-brain barrier tight junction permeability and ischemic,” Neurobiol. Dis. **32**(2), 200–219 (2008). Copyright 2008 Elsevier.

In acute brain injuries such as neonatal HIE, pediatric or adult stroke or TBI, the goal is often to treat as soon as possible. In these instances, it is important to think about how physiology will affect delivery of a therapeutic. The molecular cascades that occur after a traumatic event to the brain, independent of etiology, are complex, time-dependent, and multifocal **(**Fig. 3).^100^

Acutely after brain injury, molecular changes in the neurovascular unit (NVU) result in the formation of a permeable endothelium and loss of blood-brain barrier (BBB) integrity across multiple phases^101^ that leads to cerebral edema. Edema alters fluid distribution and diffusion within the brain, which can impact therapeutic distribution.^102^ Edema fluid spreads via bulk flow through the brain parenchyma, driven by osmotic and hydrostatic forces that are caused by a mass disruption of ion transport.^103^ If timed appropriately such as to capture this early edema phase, any movement of a therapeutic “suspended” in the fluid, either from blood or cerebro-spinal fluid (CSF), would be dictated by the bulk movement of the fluid. Mechanistic studies *in vivo* in rodents have shown that edema results in a reduction of the extracellular space (ECS) volume (due to cell swelling), which produces a marked decrease in diffusive capability of any substance in the extracellular fluid.^104–106^ An inability to effectively diffuse in the brain ECS is associated with poor therapeutic outcomes for drug and gene delivery applications.^107–109^ Edema can therefore significantly affect therapeutic impact if therapeutic uptake is diverted, slowed, altered, or prohibited by ECS changes. Interestingly, for therapeutics targeted to specific cell types in the brain, different cells undergo cellular edema at slightly different times following injury. In ischemia, astrocytes are the first to swell, even within 5 min of interrupted energy supply, which persists for 24 h.^110^ Neurons and oligodendrocytes swell as early as 30 min after injury, and become necrotic within 12–24 h.^111^ Thus, the effect of edema on ECS and cellular volume, and targeted cellular uptake, should be taken into consideration when timing the administration of a therapeutic.

Adenosine triphosphate depletion is also an immediate problem in acute brain injury. It is difficult to address primary energy failure due to loss of cerebral oxygenation and blood flow during the insult, since this requires knowledge of the timing at which the injury will occur. Therefore, beyond preconditioning-focused strategies, many therapies are targeted to attenuate inflammation, oxidative stress, or excitotoxic cascades to reduce apoptosis in the secondary injury phase. In this scenario, timing is critical. A therapy targeted at initiating recovery or repair is going to need energy to meet the demand of the intended response to treatment. If timed when mitochondrial permeabilization is under way, there is indication that recovery of the brain may be past the point of no return.^112^ The importance of timing treatment in the intricate balance of energy requirement vs availability is well documented in the neonatal HIE field using TH,^113^ particularly the timing around the onset of seizures, a common neurological outcome following neonatal HIE. Additionally, therapies targeted toward glia might need to better account for the variable energy demands of these cells. For instance, the energy demand from microglia, which are the main tissue-resident responders to any injurious event, could determine how and when these cells react to, mediate, or propagate different pathological conditions.^114^

### B. Brain environment changes that exist on a continuum

Many cellular, structural, and molecular changes in the brain following injury or development of disease exist on a continuum. In response to acute trauma, BBB disruption is temporally dependent and heterogeneous depending on the severity of the disruption, extent of permeability, and duration of the BBB opening. Given that the BBB is the most-cited reason for failed therapeutic effects in the brain, the timing and extent of this barriers' functional breakdown are important to understand for engineering therapeutic interventions. BBB breakdown can be continuous, monophasic, or biphasic after stroke, TBI, and acute spinal cord injury, especially when reperfusion is a part of the injury response.^115,116^ The impairment of the BBB is also evident in chronic neurological diseases such as AD and MS, with parallel barrier disruption seen in the spinal cord of patients with ALS.^117–121^ The major concern regarding BBB dysfunction in both acute and chronic neurological disorders is the likelihood of ongoing exposures that may contribute to or exacerbate the underlying injury. Increased BBB permeability is associated with a number of blood-borne components that can be directly or indirectly neurotoxic, via inflammation, oxidative stress, or disruption of the extracellular matrix (ECM).^122^ Therefore, in the setting of disease-directed engineering, leveraging or targeting BBB dysfunction will remain an intricate balance. BBB permeability may often be an important contributor to therapeutic efficacy, but increased permeability of the BBB may itself prevent the resolution of disease despite other targeted therapies. Improving the BBB function without identification of the root causes of neurological damage is also unlikely to provide significant long-term disease improvements.

After an acute injury, changes in cell composition and function, and the associated disruption of the ECM are important aspects of the cycle of BBB breakdown, edema, and energy depletion discussed previously. The progression of chronic neurodegenerative diseases frequently involves frank cellular loss from within the central nervous system (CNS). The absence of these cells is likely to dramatically alter the ECS volume as well as the diffusivity of extracellular fluid around the remaining structures. The losses in white and gray matter, alongside increases in local diffusivity and BBB impairment, may allow for improved access of a drug to a target site by improving capacity for diffusion. In addition to cellular loss, changes in cellularity, particularly cellular proliferation and scarring, are likely to negatively affect the ability of a therapeutic to reach its intended target. Microglia and astrocytes become activated and there is an influx of cells from the blood or periphery that are recruited by cytokines, adhesion molecules, and chemokines across the blood vessel wall. What has become increasingly apparent in the last few decades of research into the role of microglia and astrocytes is that these cell types can play both pathogenic and protective roles. This is etiology-, timing-, species-, and age-dependent, but can significantly influence the treatment effect when inflammation is a target.^123–125^

Alongside the loss or alteration of specific cell populations within the brain, both intracellular and extracellular changes in the protein composition and aggregation are seen.^126–129^ Similar to the changes in the cell function and phenotype, ECM changes can have positive and negative consequences for functional recovery in the brain. Degradation of the ECM network following injury is often followed by synthesis and deposition of newly expressed ECM molecules. Although the mechanism and timing of these changes are not well-understood in acute disease etiologies, there is strong evidence to support the effect the ECM has on therapeutic penetration of nanoparticles, modified drugs, free drugs, and cells.^130–132^ Alterations in local fluid viscosity from increased protein levels and fragmentation can decrease therapeutic movement though this space. Changes in spacing in the ECM “mesh” can also alter the ability of a therapeutic to navigate this environment.^133,134^ Additionally, accumulation of intracellular and extracellular proteins or protein plaques, as seen in many neurological diseases, may alter the capacity of a therapeutic to diffuse within the ECM or the cytosol and reach its target site. However, the way in which aggregation of proteins in the ECS interacts with cellular losses and/or changes in local diffusion to alter access of therapeutics is yet to be determined. Therapeutics will need to codeliver a drug to favorably alter the ECM for increased penetration, or be designed to navigate the ECM with minimal off-target interactions.

### C. Chronic neurological disease

Local and systemic pathophysiological processes are present in chronic neurodegenerative conditions that may alter response to therapy, or which may prevent resolution of a disease process if not actively targeted alongside a neurotherapeutic. Delivery of a therapeutic, and providing the necessary conditions for cellular recovery and repair, requires appropriate and regulated CBF. In the healthy brain, this is achieved through neurovascular coupling (NVC) via the NVU.^122^ A fully functional NVU responds to increasing metabolic demands within an active area of the brain by providing additional blood flow and with it, increased delivery of oxygen and metabolic fuels (i.e., glucose, ketone bodies, or lactate). Abnormal NVC is commonly seen, including in AD and chronic mild TBI or postconcussive syndrome (PCS).^135–138^ Another hallmark of chronic neurological disease is the disruption of normal sleep patterns, which can act as both a cause and consequence of neurodegeneration, particularly in dementia and AD. Though the mechanisms are not entirely understood, sleep is thought to represent a crucial period of metabolic waste clearance from the brain, potentially due to the increased volume of, and flux through, the glymphatic system.^139^ Ensuring adequate sleep support, either through environmental, cognitive, behavioral, or pharmacological means, could therefore be a crucial determinant of the success of therapeutics for a wide range of neurological disorders.^140,141^

### D. Recommendations and cautions for leveraging changes in the brain environment

Physiological changes at or close to the site of active injury or inflammation do have the potential to enhance uptake, and increase delivery, of therapeutics to the desired target. This effect has been leveraged in multiple preclinical studies of neuroinflammation-mediated brain injury, where BBB permeability measured by permeation of a dextran or Evans Blue albumin results in associated increased therapeutic uptake compared to healthy or sham controls.^142–144^ Due to this potential for delivery, the extent of BBB opening or leakiness has been of keen interest to therapeutic delivery strategies. In some injury models, there is time-dependent passage of small and large molecules across the impaired BBB. In the immediate hours after TBI, both large and small molecules have been demonstrated to transport across the BBB.^145^ However, within 4–5 h after TBI, large molecule transport becomes restricted, until 2–3 days later.^146,147^

The degree of BBB disruption does influence size-dependent extravasation. Interestingly, milder BBB disruptions appear to be associated with transcellular pathways and allow movement of small molecules, whereas severe disruptions are mediated by paracellular loss of tight junctions, which allows movement of larger molecules. Yet it should be noted that the permeability of the BBB is variable depending upon the imaging methods and extravasating dyes used.^148^ Engineering therapeutic delivery systems should utilize the right sized agent during the right window of opportunity. Additionally, the brain drug delivery field is fraught with inconsistencies in what it means for a therapeutic to bypass the BBB and in the implementation of methods to assess the extent of passage across the BBB.^149^ Many studies measure the percent injected dose of a drug in the brain by extracting the brain, homogenizing the brain tissue, and isolating the therapeutic agent from the homogenate. There are several potential issues with this approach. Quantification of therapeutic levels in the homogenate does not equate penetration of a therapeutic into the brain parenchyma or extravasation away from the NVU. Perfusing vasculature prior to brain extraction can increase the likelihood that the measured therapeutic values in the homogenate are more representative of therapeutic penetration in tissue and not of the amount in the luminal space. However, perfusion methods should be rigorously performed and controlled,^150^ and there are few and limited studies that have evaluated how perfusion directly affects the distribution of engineered therapeutics within the luminal space. More sensitive quantification can be achieved with capillary depletion techniques,^151^ where the vasculature is isolated from the parenchyma and quantified separately. While generally considered robust, these studies are time dependent, and depending on the rate at which a therapeutic interacts with endothelial cells or receptors, redistribution of a therapeutic can happen.^152^ It can therefore be important to include time-dependent quantitative imaging analysis that captures the spatial distribution of therapeutic platforms, to identify region specific uptake and extravasation away from the NVU.

### E. Pitfalls in modeling disease etiology and progression

Most diseases modeled in the preclinical setting do not occur spontaneously in laboratory animals in a predictable or standardized manner, so they must therefore be initiated through some acute process or genetic manipulation. In preclinical models, the initiation and etiology of disease or injury therefore tends to be known, and can be directly or immediately investigated and targeted. By contrast, in both the acute and chronic disease setting in humans, there are barriers to translating preclinical work into effective therapies. In humans with acute injury, the timing of the injury is often known, but access and timing of therapy is challenging, such as timely and optimal treatment of stroke or TBI.^153,154^ This is particularly true at the clinical trial stage when consent of the patient, imaging to confirm diagnosis, and proximity to a trauma center or stroke center, for example, are critical. In human neonates born with some degree of brain injury, both preterm and term, there is often some unknown period that includes *in utero* infection or intermittent HI before birth occurs,^155^ and therefore, the exact timing or direct cause of the injury is uncertain. Similarly, in the setting of chronic neurological diseases, the causative factors and timing of the exposure are generally not considered, either because they are not known, or because the focus is on treating the downstream effects rather than the upstream initiating factors.

Improving the identification of acute neurological injury and improving the timing of treatment is likely to continue to improve the translation of preclinical therapies to the patient.^156^ However, this will still require significant improvements in the quality of how preclinical work is performed and reported, in order to ensure the most promising therapies are put forward for clinical trials. Suggested methodological improvements include those outlined by the ARRIVE (animal research: reporting of *in vivo* experiments) guidelines and STAIR (Stroke Therapy Academic Industry Roundtable), the latter of which were developed particularly to aid in the translation of therapies for acute stroke.^157,158^ These guidelines have been in existence for at least a decade but are still not being consistently used by preclinical investigators, or enforced by journals.^157^ Similarly, it should be noted that any therapy being investigated for treatment of acute neurological injury be assessed for its effects on core temperature and thermoregulation.^159^ As TH is the most consistently beneficial preclinical therapy for acute neurological injury across species and ages, and multiple drugs directly affect thermoregulation, it is interesting to note that reassessments of previous therapies suggests they are mainly beneficial due to their effects on temperature.^159^ Despite this, longitudinal measurement of core temperature after injury and treatment is not routinely performed, even when modeling injures where TH is standard of care in the clinic.^160^ This is one crucial aspect where understanding the physiology of the disease will ensure that promising therapies have the greatest chance of success in the clinic.

In the chronic disease setting, the timing and nature of the insult are generally not known, and therefore, it is the downstream processes that are modeled preclinically, rather than the initiating factors themselves. This is particularly prevalent in AD as well as MS, where the most common preclinical model used is experimental autoimmune encephalomyelitis (EAE). The EAE model of MS is largely used in mice, and involves inoculating the animal with CNS antigens including whole CNS homogenate, proteolipid protein (PLP), myelin basic protein (MBP), or myelin oligodendrocyte protein (MOG), in an emulsion with an adjuvant.^161^ This results in an autoimmune response that includes CNS demyelination, and is thought to recapitulate some of the immunologic and pathologic processes seen in MS. Though most of the disease modifying drugs that have translated to clinical use were successfully employed in the EAE model, there is yet to be an MS drug that significantly alters the long-term progression and mortality of the disease.^162^ This is at least partly due to the fact that the EAE model does not truly investigate the disease pathogenesis, as is usually suggested. For this to be the case, a model would include the natural history and environmental exposures that are associated with MS risk and disease progression. Instead, the EAE model mimics some of the processes that may occur after the peripheral immune system is exposed to CNS antigens.^161^ To truly model and understand diseases like human MS, one should ask what it is that causes the initial damage to white matter (modeled by injecting CNS antigens in EAE), loss of the relative immune privilege of the brain (including BBB permeability), and immune activation (modeled by adjuvants in EAE), such that a systemic autoimmune response resulting in demyelinating lesions occurs.

Similar problems exist in preclinical models of AD. In order to target the accumulation of Aβ or hyperphosphorylated tau, under the assumption that these are the etiologic targets of the disease process, transgenic mouse models that overexpress these proteins have become a mainstay of preclinical AD research.^163^ However, these models have failed to result in successful clinical trial outcomes, and in a similar manner to MS models of EAE, this could be due to the lack of focus on disease etiology. Rather than focusing on how to clear Aβ plaques and tau neurofibrillary tangles once they have accumulated, we may see better outcomes by investigating the wide range of exposures that cause these proteins to accumulate in the first place. In a wide range of animal models of neurological disease, by focusing on a single downstream pathological process, we have made it almost impossible to ascertain or investigate the crucial upstream processes that initiate the disease in the first place. This is likely why these animal models are unable to model crucial aspects of the human pathophysiology.

These problems are consistently seen across the spectrum of preclinical models for chronic neurological diseases—this is true in the areas of ALS and ASD research, as well. Importantly, and relevant to any model resulting from genetic manipulation, there is relatively little known about whole-network effects of a specific genetic knockout or knockdown. Depending on how an alteration within a specific gene occurs, other aspects of metabolism may or may not be able to compensate.^164^ Similarly, the method of genetic manipulation, including the increasingly popular CRISPR–Cas9 system, may result in significant deleterious effects outside of the gene of interest.^165^ There may also be off-target effects that alter the resulting phenotype, and if the entire transcriptome, proteome, and metabolome is not characterized, these effects may be ascribed to the gene itself rather than to other genetic or epigenetic changes. It is also worth noting that genetic and phenotypic evolution can occur very rapidly, in some cases within 10 generations.^166,167^ This suggests that rapid changes can occur within one animal colony based on both genetics and environment, resulting in adaptations to a particular genetic manipulation that are almost impossible to predict and must be regularly and fully characterized in order for the resulting data to be interpreted.

While we certainly appreciate the significant complexity involved in developing and maintaining animal models of neurological disease, these limitations of the current approaches must be addressed, or at least controlled for, if we are to improve the translation of therapies to the clinic. A physiology-driven approach, especially to chronic neurological disease, must therefore include an understanding of what causes a disease in the first place, rather than simply focusing on downstream processes by creating an artificial genetic manipulation or acute stimulatory event. This is why we believe an engineering approach to disease treatment is crucial. Though it is an oversimplification, engineers are trained to understand why the failure of a system occurred in the first place in order to prevent it from happening again, rather than simply focusing on how to fix the problem once it has occurred.

## IV. IMPORTANCE OF INCORPORATING SYSTEMIC PHYSIOLOGY AND TRUE DISEASE ETIOLOGY

In addition to normal physiological processes that differ across the biological areas, the importance of acknowledging the systemic physiological changes that might be present, even if a treatment intervention is targeted toward a local pathophysiological setting, must also be emphasized.

### A. Systemic physiology vs local physiology

Increasing evidence suggests that an integrated approach to the treatment of both acute and chronic neurological disease is likely to require an understanding of systemic physiology. This is especially true for the peripheral immune and gastrointestinal systems, particularly in the case of oral and other routes of systemic administration of engineered therapeutics. The physiology and microbiota of the gut appear to play an important role in the development of a number of neurological disorders. The gut microbiota has been implicated in the etiology of chronic neurodegenerative conditions such as PD and AD, as well as MS and ALS.^168–170^ The nervous system can also directly affect gut physiology. Activity of the vagus nerve may decrease systemic inflammation, as well as have a permissive effect on normal gastrointestinal function.^171,172^ Acute neurological injury appears to negatively affect the gut function as well. TBI in a number of animal models, and in humans, results in increased intestinal permeability due to the loss of tight junction function.^173^ This leads to increased bacterial translocation and systemic inflammation, which contributes to the ongoing injury process.^173–175^ When considering therapeutic approaches, it is worth noting that bacterial endotoxins also appear to alter hepatic function and xenobiotic metabolism.^176^ This suggests that considering the systemic and peripheral responses to injury, including on gut function and how that may affect drug uptake or metabolism, must be taken into account when designing therapeutics for acute neurological injury. For chronic neurological conditions, the gut microbiota and other environmental aspects of patient lifestyle are likely to provide further complexity.^177–179^

The endocrine and autonomic nervous systems are key drivers of systemic physiology that require normal CNS function, but can also directly affect the ability of the brain to resist or repair injury. Pituitary hypofunction and failure are commonly described after TBI, where delayed hormonal deficiencies often manifest in the months after injury.^180,181^ Depending on the study, the effects of these injuries on the gonadal, adrenal, and thyroid hormonal axes have all been described.^180,181^ The changes to these axes become particularly important when considering the importance of these hormones on neurological outcomes.^182^ In most neurodegenerative disorders, the sex hormones testosterone, estradiol, and progesterone all appear to be involved in cognition and quality of life, as well as being mechanistically associated with cell survival and repair within the CNS.^182^ Significant data from the preclinical literature led to progesterone being investigated as a neuroprotective agent after TBI, with interest also in the stroke field, though results have so far been negative.^183,184^ Estradiol is also considered a trophic factor after neurological injury, including anti-inflammatory and neurogenic properties in preclinical models.^185,186^ In men particularly, though not exclusively,^187^ low testosterone is associated with worsening cognition and depressive symptoms, and testosterone replacement appears to improve cognition and depressive symptoms in those with low testosterone in an inverted U-shaped manner.^188^ However, those with both cognitive decline and low testosterone do not seem to benefit, likely due to the fact that low testosterone is not the cause of the cognitive decline per se, but rather that the two are affected by a common underlying disease process.

As well as being critical for normal neurodevelopment, thyroid hormones are also essential for normal adult brain function.^189^ Both low and high thyroid stimulating hormone release from the pituitary are associated with an increased risk of cognitive decline and AD.^190,191^ Similarly, both low and high circulating thyroid hormones are associated with worse neurological outcomes. However, thyroid replacement in older adults with subclinical hypothyroidism does not improve cognition.^192^ Overall, a certain amount of thyroid hormone is almost certainly required for adequate metabolic rate and mitochondrial function, but elevated levels may result in increased metabolic demand in the face of reduced or disordered nutrient supply. Similar to sex hormones and neurological outcomes, ensuring adequate presence of thyroid hormones is important, but replacement without addressing underlying disease processes is unlikely to provide further improvement.

From the standpoint of ensuring the maximum potential success from a neurotherapeutic, the goldilocks effects of the major hormones must be considered—disordered hormone levels can be both a consequence of neurodegenerative diseases as well as a necessary factor for optimal neurological function. For both thyroid and sex hormones, careful replacement in those with low levels can be important for both disease outcomes and quality of life, as well as potentially for the success of a therapeutic intervention, either directly due to being necessary for recovery, or due to a deficiency confounding an outcome variable, such as cognitive function. However, it is important to remember that there are associated increased risks of cardiovascular disease with sex hormone and thyroid hormone replacement, as well as certain cancers, if not performed carefully.^193–195^

### B. Correlation vs causality

One significant, yet crucial, aspect to investigating the pathophysiology of (neurological) disease, and subsequent therapeutic targets, is discerning mechanisms that can be considered a root cause for treatment from epiphenomenal biomarkers of an underlying disease process. One of the most noticeable examples in neuroscience research is the amyloid cascade hypothesis (ACH), and the identification and targeting of amyloid-beta (Aβ) plaques in the treatment of AD.^196^ As mentioned briefly above, despite decades refining and examining the ACH, a number of high-profile clinical trial failures suggest that drugs (such as enzyme inhibitors and monoclonal antibodies) targeting both the production and accumulation of Aβ have no impact on long-term outcomes in AD.^197^ Though there remains genetic evidence that Aβ accumulation may be a component AD pathophysiology,^198^ and some suggest that earlier drug timing in relation to disease progression may be the key to targeting Aβ in AD,^199^ there are an increasing number of voices suggesting that a broader focus must now be taken.^200–202^ Even when examining the connection between Aβ plaques and severity and progression of symptoms in AD, the evidence is considered weak.^201^ Furthermore, there is a significant growing burden of evidence that Aβ accumulation represents a response to neuroinflammation or toxic exposures, and Aβ may have antioxidant and antimicrobial effects.^203–205^ This growing evidence suggests that AD and cognitive decline is a downstream result of various environmental exposures, with Aβ plaque accumulation representing part of the final common pathway of neuronal injury.^206^ Though significant Aβ accumulation may in itself become damaging,^207^ Aβ is much more likely to be an epiphenomenal biomarker of neuronal stress, and perhaps even a protective response, rather than a therapeutic target. The targeting of Aβ accumulation itself, as well as the increasing focus on downstream accumulation of Tau protein in neurofibrillary tangles, should perhaps be reconsidered in favor of the identification and understanding of the causes of neuronal stress and inflammation. Even if the ACH is correct, the fact that there remains significant debate as to its veracity indicates the need for a broader investigation of the physiology underlying AD. More broadly, this suggests that engineering therapeutics to target singular molecular processes without incorporating the context of the greater disease and physiological environment is a strategy that is unlikely to result in translatable therapeutics, with many failed examples already available in the neuroscience field.

## V. HIGH LEVEL DISEASE MODULATORS AND INTERVENTIONS WHEN TRANSLATING TO THE CLINIC

**FIG. 4.**
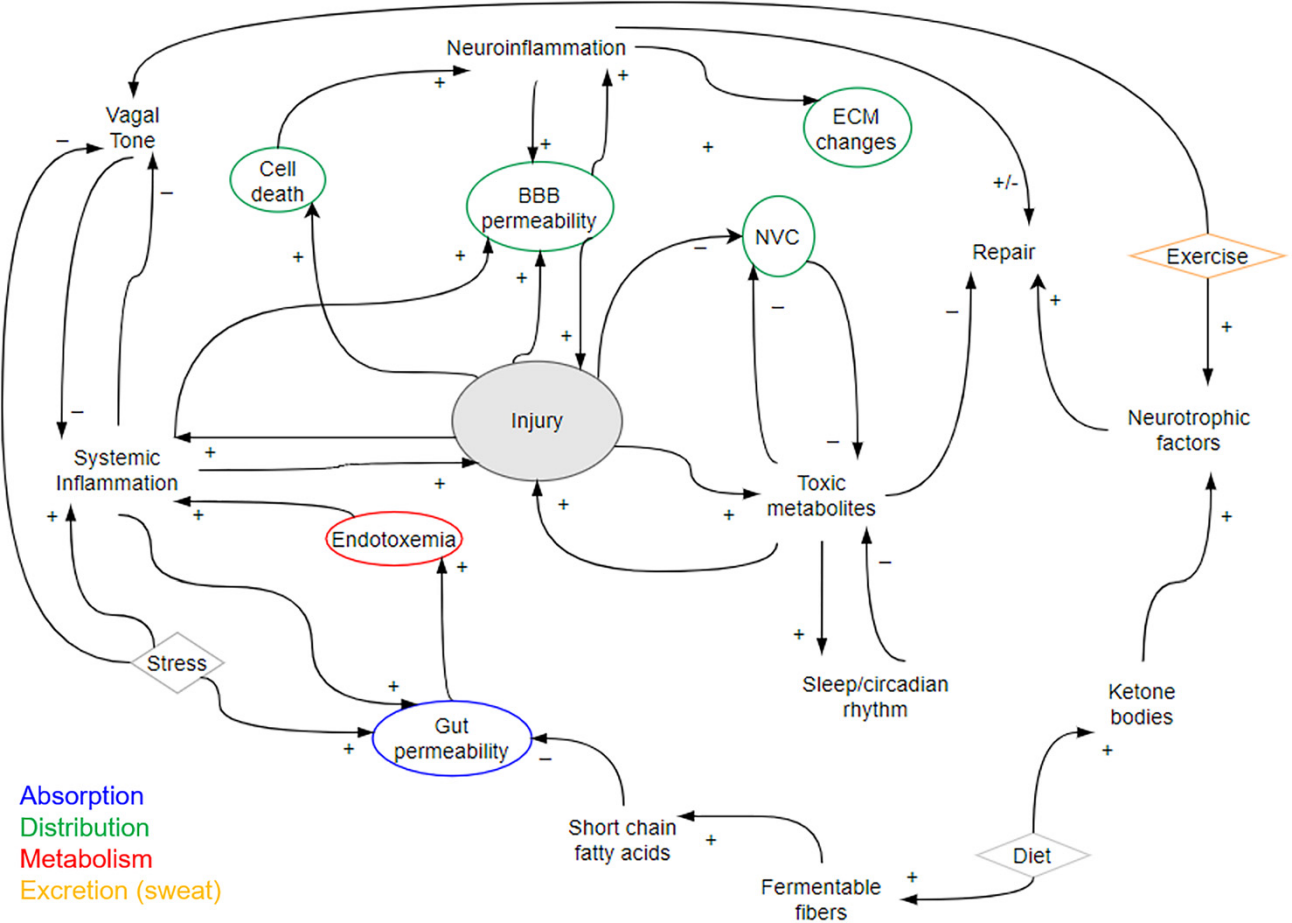
Causal loop diagram of pathophysiological changes in neurological disease. The diagram highlights some of the factors such as sleep, diet, and exercise that could modulate, positively (+) or negatively (−), aspects of neurological disease. In addition, factors that would affect the absorption (blue), distribution (green), metabolism (red), and excretion (orange) of therapeutics in the context of a pathophysiological state are outlined. Single aspects of how high-level interventions such as diet might affect disease are used as examples. Normal hormonal and autonomic physiology also has a permissive effect on both local and systemic injury and recovery.

Pertinent to any discussion on trying to reverse disease and develop therapeutics is the myriad lifestyle and environmental factors that have been shown to improve long-term neurological disease burden and patient outcomes. Many of these interventions, including sleep, diet, and exercise, have significant epidemiological, and increasing clinical evidence to support their use in the long-term treatment of neurological diseases, despite the fact that the mechanisms underlying their benefit have yet to be fully elucidated. Indeed, their benefit may lie in the fact that they affect multiple pathways and disease risk mechanisms rather than relying on an intervention at a single mechanistic point, which is an approach that has generally failed in the treatment of spontaneous neurological disorders and chronic disease.^208^ “High-level” interventions are increasingly being incorporated into long-term “holistic” care initiatives for those with neurological injury, but may also be adopted by patients themselves outside of a formal care plan. As such, if these interventions are not adequately accounted for, they may also interfere with the outcomes of therapeutic trials. High-level strategies to improve outcomes in neurological disease include sleep, dietary changes, and exercise and rehabilitation. The effects of socioeconomic status and social interactions also have the potential to dramatically change neurological outcomes regardless of the initial severity of disease. Though the in-depth discussion of these factors is beyond the scope of this review, a potential incorporation of these factors in modulating pathophysiological aspects of neurological disease are provided in Fig. 4.

A combined model that incorporates high level factors in addition to disease-specific mechanisms and responses to therapy might suggest that three broad things are required for optimal neurological recovery, repair, and function after an injury: (i) the presence of necessary nutrients, hormones, and oxygen (via the NVU), (ii) the absence of neurotoxic substances including heavy metals, excess cortisol, blood-borne proteins, and accumulated Aβ and tau, and (iii) a healthy environment including the ECM and a functional BBB, but also a broader supportive social environment for the person themselves. Without ensuring that each of these factors is addressed, the poor translation of therapies to the clinic may continue. Though robust clinical trials are lacking, multipronged physiological approaches to disease etiology have been found to be beneficial, for instance resulting in a reversal of cognitive decline in a number of case studies of AD, and in mood and executive function in MS.^209–212^ In reality, the best long-term outcomes are likely to involve multiple interventions. If the patient's best interests are at heart, multiple interventions may (for the time being at least) be implemented without knowing exactly how or how much each one contributes to the final outcome. This highlights some of the downsides of the traditional randomized controlled trial (RCT) structure when incorporating multiple approaches into a single therapy. Factorial trials could implement dietary-, exercise-, sleep-, and pharmaceutical-based interventions in multiple combinations, but this would require a large number of patients and significant clinical trial infrastructure. However, some assistance could be provided by the rapidly developing state-of-the-art in digital health approaches that utilize self-monitoring and automated smartphone apps to both monitor and personalize lifestyle-based interventions, as well as increase adherence, for instance through just-in-time adaptive interventions.^213^ Another alternative from the behavioral science arena could be to test multipronged interventions, including pharmaceutical therapies, using a control optimization trial (COT) structure. The development of COTs comes from control systems engineering,^214^ and focuses on a single output that can be regularly and easily monitored, such as cognitive functions tests in AD or after TBI. Using a “small data” approach,^215^ holistic assessment of the patient, personalized trajectories for hormonal, sleep, dietary, or pharmaceutical intervention could be applied in an order determined by the likely “lowest hanging fruit” for each patient, with weekly cognitive function testing being the factor being optimized for. Any significant improvement or deterioration with a step-change in therapy (or other event) could be rapidly identified and acted upon (if necessary), rather than waiting for the outcomes of a six-month static intervention as might be dictated by the structure of a traditional RCT.

## VI. AN EXAMPLE OF SUCCESS IN PHYSIOLOGY-DRIVEN TREATMENT INTERVENTION FOR BRAIN

As mentioned above, the neonatal neuroscience field stands out with regard to physiology-driven interventions as the physiology is often very well-described, and therapies are tested in several species and models before being translated to clinical trials. For the rapidly growing field of nanotechnology-based therapeutics, the integration of nanoparticle design with the physiology of the brain and target disease is crucial for success.^131,216^ Specifically, the barriers to delivery in the brain must be accounted for collectively, as well as in the context of the disease. To be successful, a nanoparticle must overcome or traverse the BBB, penetrate the brain ECS, and have action on or uptake into specific disease-associated cells, leaving healthy cells alone.^131^ Here, we will focus on the successful translation of an engineered dendrimer nanoparticle platform. A dendrimer-drug platform has now made its way into clinical trials in the difficult-to-translate pediatric population, based on comprehensive assessment of the ability to overcome barriers to delivery to the brain in multiple species and multiple models of inflammation-mediated brain injury.

Dendrimer nanoparticles are globular, repeatedly branched, macromolecular structures with high tunability and tailorability, on the size scale of 3–10 nm. Dendrimer nanoparticles have shown promise in treatment of cancer, systemic inflammation, and ocular disease.^217,218^ Polyamidoamine (PAMAM) dendrimers, in particular, have also been extensively studied and evaluated in inflammation-mediated brain diseases, particularly diseases that affect the neonatal and pediatric populations. In developing a dendrimer-drug for treatment of newborn brain disease, many design aspects were considered in the context of the physiology of the disease. As described in Sec. III, BBB disruption, cellular activation, and ECM alterations occur in acute brain injury and in neurodevelopmental disorders. These changes can be leveraged through designing nanoparticle physicochemical properties and characterizing pathophysiology to accomplish effective uptake and site-specific targeting of nanotherapeutics.

Nanoparticle physicochemical properties can be tailored to maximally overcome disease-related physiological changes. The role of dendrimer size and surface charge in pharmacokinetics (PK) and biodistribution has been explored in a pediatric context.^219–221^ Generation 4 hydroxyl-modified (G4-OH) PAMAM dendrimers, 4 nm in size, have short circulation half-lives on the order of 30 min to 1 h, but readily uptake into the brain of diseased neonatal animals after intravenous administration.^219^ Increasing dendrimer size to generation 6 can increase circulation time and uptake in the injured brain.^222^ However, for brain uptake to occur, the BBB must be disrupted. The presence and extent of BBB disruption after the initiation of the disease strongly correlate with dendrimer uptake.^220^ Once across the BBB, the use of G4-OH dendrimers allows for diffusion and penetration with the brain extracellular space, increasing distribution and access to disease microglial cells.^220^ The hydroxyl functionality is critical—intravenous administration of amine-modified dendrimers, even in regions where the BBB is impaired, does not extravasate into brain parenchyma. Direct parenchymal injection of amine-modified dendrimers of the same size does not penetrate the brain ECS or uptake into microglial cells.^220^ The use of carboxyl functionality results in delayed extravasation into the brain parenchyma, and a more punctuated intracellular distribution^223^ in microglial cells compared to hydroxyl-modified platforms. The ability to maximally distribute based on control of surface charge was also demonstrated in a glioma model^224^ and following intra-amniotic administration.^221^

The increased access to microglia cells is particularly critical for therapeutics geared toward attenuating inflammation. A growing body of literature demonstrates that nanoparticles, including G4-OH PAMAM dendrimers, have the ability to rapidly uptake in activated microglia in the injured brain in regions of injury.^93,142,143,217,220–222,225–229^ The uptake of a dendrimer-drug conjugate into activated microglia has resulted in improvement in neurological outcome in a variety of models, highlighted in Table I. Although microglia are the dominate cell-type for PAMAM dendrimers to accumulate in, cell-specific uptake in astrocytes, neurons, and oligodendrocytes is dependent on etiology and timing of administration.^93,142,227^

**TABLE I.** Summary of dendrimer-NAC efficacy studies for treatment of inflammation-mediated injury. PAMAM dendrimers conjugated with NAC (D-NAC) have been tested in a variety of animal models and species. The table provides the disease model and corresponding phenotype, species including strain where relevant, etiology of the disease, administration route and frequency of the D-NAC, and primary outcomes related to efficacy. Additional efficacy studies utilizing PAMAM dendrimer platform with drugs other than NAC, and in different disease models, are reviewed elsewhere.^216^

Disease model	Species	Etiology	Clinical phenotype	Administration route	Outcomes	References
Brain-specific injury or disease						
Maternal inflammation-mediated cerebral palsy	Rabbit	Intrauterine lipopolysaccharide (LPS) administration at gestation day (G) 28	Cerebral palsy (CP)	Single intravenous on P1	Selective localization in activated microglia and astrocytes in the brain of newborn rabbits with CP, but not healthy controls; suppressed neuroinflammation; dramatic improvement in motor function in the CP kits	143
Ischemic white matter injury	Mouse (CD-1)	Unilateral carotid artery ligation at postnatal day (P) 5	Periventricular leukomalacia (PVL)	Single intraperitoneal on P6 or P10	Sustained attenuation of the “detrimental” proinflammatory response up to 9 days after injury, while not impacting the “favorable” antiinflammatory response; improvement in myelination, suggesting reduced white matter injury	142
Rett syndrome	Mouse (C57B/6)	Knockout of the mecp2 gene	Rett syndrome	Intraperitoneal twice weekly	Localization in microglia in Mecp2-null mice, but not in age-matched wild type littermates; significant improvement in behavioral outcomes in Mecp2-null mice, but not in survival.	226
Hypothermic cardiac arrest	Dog	Closed-chest cardio-pulmonary bypass followed by cooling (to 18 °C), hypothermic cardiac arrest for 2 h	Cardiac arrest-induced brain injury	Single intravenous bolus infusion	Combination therapy with D-NAC and D-VPA showed produced 24 h neurological deficit score improvements at one-tenth the dose of free drug; significantly reduced adverse side effects	93
Hypoxia-ischemia	Mouse (CD-1)	Unilateral carotid artery ligation on P7 followed by hypoxia	Neonatal HIE	Single intraperitoneal dose on P7 or P8	Uptake correlated with brain injury in all cell types; uptake was not inhibited by hypothermia, except in CD68+ microglia; targeting of microglia, astrocytes and neurons was achieved	227
Other models where inflammation plays a mediating role						
Intrauterine inflammation	Mouse	Intrauterine LPS on embryonic day (E) 17	Preterm birth	Single maternal intraperitoneal	Significant reduction in preterm birth rate; altered placental immune profile with decreased CD8+ T-cell infiltration; improved neurobehavioral outcomes; reduced fetal neuroinflammation and long-term microglial activation in offspring	225
Adreno-leukodystrophy (ALD)	Human monocytes	Healthy, heterozygote carrier, adrenomyeloneuropathy, and cerebral ALD patient-derived cells	X-linked ALD	Topical (*in vitro*)	Dose-dependent reduction in TNFα and glutamate secretion; replenished total intracellular glutathione levels in cALD patient macrophages	229
Necrotizing endocolitis (NEC)	Mouse (C57B/6 with TLR4 knockout)	Gavage feeding of formula with enteric bacteria isolated from an infant with NEC.	NEC and NEC-induced brain injury	Oral administration	Prevention of NEC-associated neurological dysfunction in neonatal mice	228

Based on this body of comprehensive and extensive preclinical work for dendrimer-*N*-acetyl cysteine (NAC), the FDA approved the investigation of this platform in humans. A PAMAM dendrimer platform conjugated with *N*-acetyl cysteine (NAC) is currently being investigated in a Phase I study for safety, tolerability, and PK,^230^ for next Phase use in patients with cerebral adrenal leukodystrophy, a rare and fatal orphan disease. The success of translating a nanoparticle platform into the pediatric population is significant, and has never been previously achieved for a noncancer indication. Yet the process to achieving this was no small feat and highlights the importance of using disease physiology to guide treatment intervention and success. Additionally, the path of translation of this particular dendrimer-drug conjugate leveraged multiple animal models of inflammation-mediated disease, accounting for sex differences and developmentally appropriate ages for each model. The use of multiple disease models in multiple species is an important feature of this translational work; given that the etiology of neurodevelopmental diseases is complex, and the progression of a disease is heterogenous from patient to patient, evaluation of a therapeutic platform in multiple models with hallmarks of disease better replicates the real-world clinical setting.

## VII. CONCLUSIONS AND THE PATH FORWARD

In 2002, Yuri Lazebnik applied the current reductionist and mechanistic approach generally seen in the fields of biology to a known complex engineering problem (fixing a broken radio), and elegantly described why the biologist's approach would be likely to fail.^208^ While not much has changed, either in terms of approach or success in the field of neuroscience in nearly two decades since, we are encouraged by the fact that engineering has recently played a larger role in describing, modeling, and quantifying biology to further the understanding of therapeutic discovery and development.

There remain many challenges and opportunities to be furthered in the area of improving drug delivery for neurological disease.^231^ Neurological diseases are a complex domain that cannot be reduced to mere molecular pathways to identify potential treatments or cures. We must acknowledge this complexity, and continue to develop therapeutic technologies that address the breadth and subtlety of disease. Yet, we cannot successfully do this in a time and cost-efficient manner without letting the disease physiology direct our engineering of a therapeutic intervention. Thus, our goal in this perspective has been to highlight the fact that translation of therapeutics is increasingly likely to fail if the true scope of human disease physiology is not considered or captured across the range of initial preclinical work. We also highlight a number of physiological processes, both normal and pathological, that can and should be integrated into therapeutic development in order to increase the likelihood of translation. Given the complexity of accounting for all possible factors, we have highlighted several starting points in Table II that could be readily done from any biomedically focused engineering lab with necessary collaborations with basic and clinical scientists.

**TABLE II.** Proposed methods for implementation of a disease-directed engineering approach. For each section of the perspective, we provide actionable areas of emphasis that a biomedically focused engineering lab could implement to follow a disease-directed engineering approach. We also provide examples of how the actionable areas could be carried out in the lab setting. Where we felt most relevant, we emphasized the critical need for collaborations.

Implementation of a disease-directed engineering approach	Actionable areas of emphasis	Example
Normal physiology should drive experimental design	Include both sexes in all studies	Until the sex dependence of the outcome is established, therapeutic studies should be powered to account for sexual dimorphism
	Use age- and developmentally appropriate models	Evaluate therapeutics for neurodegenerative disease in older animals; or if testing a therapeutic for preterm versus term brain injury, ensure that susceptible brain structures match human development
	Evaluate in multiple species (if available) of the same disease model^a^	Therapeutic hypothermia for term HIE was shown to be successful in HI models in rats, pigs, and sheep before being translated to the clinic.
Disease physiology should direct treatment intervention	Assess hormonal and gut function via blood hormone levels (LC-MS or ELISAs) and gut permeability (histology), respectively	In experimental TBI, gut function is acutely worse and hormonal function chronically deteriorates, therefore outcomes would need to be statistically adjusted to account for these; or alternatively, therapeutic interventions could be timed for oral delivery when gut permeability is high
	Focus on multiscalar factors for outcome assessments^b^	In MS, look at the molecular and cellular level (immune response), the whole-organ level (imaging, histology), and the whole-organism level (behavior, mortality)
Timing of pathophysiological changes could determine intervention delivery success	Evaluate how delivery of a therapeutic platform is affected by pathological changes at the organ and cellular/extracellular level	Quantify distribution, diffusion, and cellular uptake at different dosing time points after disease onset to account for compensatory pathological changes that might impair (i.e., edema) or improve (i.e., BBB permeability) delivery
	Leverage pathological changes at the appropriate time after disease onset for maximal delivery	AD has chronic BBB impairment in the areas of injury or susceptibility, therefore therapeutics that are long-circulating can be engineered to take advantage of this increased permeability based on the extent and mechanism (i.e., endothelial loss or alteration in transporter expression) of impairment
Reproducibility and translation	Test in multiple models that account for different etiologies that may result in the same phenotype^a^	Cerebral palsy can result from hypoxia-ischemia, infection, or inflammation, so evaluating a therapeutic in models of these three etiologies that result in motor function loss is essential
	Reproduce experiments in multiple labs^a^	Partner with collaborators working on the same model in the same species or collaborate with someone who has the same model in a different species
	Include multiple relevant pathologies, when relevant	If performing MCAO to model adult stroke, then include etiological factors such as hypertension, obesity, diabetes

^a^ Collaboration is key to successfully implementing these measures.

^b^ For optimal translation from preclinical to clinical implementation, the multiscalar assessment would need to be performed equally in the preclinical model and in humans.

The first step that could be the “low-hanging” fruit would be to include both sexes in therapeutic studies, to determine if there are sexually dimorphic responses to a therapeutic intervention. To effectively determine if sex is a factor, it is recommended to pick at least three functional assessments that span multiple scales. For example, measuring mitochondrial function can serve as a metric that affects immune regulation, metabolic health, and integration of hormonal processes. Mitochondrial function can be assessed alongside behavior and histology or some form of cellular-based imaging to better integrate cellular processes with outcomes that are clinically meaningful. In performing these studies, the use of age- and sex-matched animals with untreated disease animals as controls can provide the baseline changes in pathological hallmarks that will influence the therapeutic outcome. As discussed in Sec. III D, therapeutic distribution should be analyzed comprehensively, using both quantitative and qualitative methods to determine the location of the therapeutic within the context of the pathological hallmarks. Appropriate leveraging of pathological processes and accurate assessment of therapeutic localization can reduce the need to overengineer a system, thus reducing the number of design controls and number of animals needed. For example, the G4-OH PAMAM dendrimer crosses an impaired BBB, penetrates the brain parenchyma, and selectively uptakes into activated glial cells. This occurs without the need for a targeting ligand for BBB penetration or cell-targeting ligand for microglial-specific uptake. If localization in brain endothelial cells is required for maximal therapeutic effect, the OH-modification could be altered to NH_2_-modification on the G4 dendrimer as a starting point, since this surface functionality showed endothelial localization and an inability to extravasate away from impaired blood vessels.^220^ However, if targeting an intracellular compartment such as the mitochondrial membrane is required for disease treatment or therapeutic effect, then the use of a targeting ligand could prove beneficial.^232^ Once sex-dependent therapeutic effect and therapeutic localization are determined, a next step would be to identify collaborators with models of the same disease or that display critical hallmarks of the disease process in other species, to determine whether pathological processes and treatment effects translate across a wide range of evolutionary divergence. This step could also result in replication across multiple labs.

An engineering approach will play a key role to future successes by incorporating a system-level view of a disease that can be analyzed as parts of a whole, in the context of relationships with other parts and with other systems that influence health, such as environment, lifestyle, genetics, and sociocultural standards. However, in order to do this, engineers must continue to push to be immersed in the biology in order to be conversant in the physiological aspects of the disease and understand enough about each component of the disease to know its contribution to the ‘operational’ aspects of the human being. As the burden of disease continues to evolve, we must adapt our approach and engineering to continually incorporate the deepening of our biological understanding. We believe that this can be done by fostering communication and collaboration between engineering and basic science, utilizing the breadth of available animal models and physiological data from diseased populations, and integrating them into therapeutic approaches that include interventions tailored to the individual and disease across multiple levels.
